# P-1811. Impact of Hemo-Alert Program on Appropriate Antimicrobial Therapy in Hospitalized Patients with Bacteremia: Results from a Quasi-experimental Study

**DOI:** 10.1093/ofid/ofae631.1974

**Published:** 2025-01-29

**Authors:** Siwakan Phuetphaichit, Witchuda Kamolvit, Surangkana Samanloh, Pinyo Rattanaumpawan

**Affiliations:** Faculty of Medicine Siriraj Hospital, Mahidol University, Bangkok, Krung Thep, Thailand; Faculty of Medicine Siriraj Hospital, Bangkok, Krung Thep, Thailand; Faculty of Medicine Siriraj Hospital, Bangkok, Krung Thep, Thailand; Faculty of Medicine Siriraj Hospital, Mahidol University, Bangkok, Krung Thep, Thailand

## Abstract

**Background:**

Hemo-Alert program is an antimicrobial stewardship strategy, designed to shorten the time to appropriate antimicrobial therapy in bacteremic patients.

**Methods:**

Eligible subjects were all hospitalized patients with at least one positive hemoculture. The standard of care included a prompt notification of positive hemoculture via a phone call. However, the final identification and susceptibility results need to be individually retrieved. The Hemo-alert program included a prompt notification of final identification and susceptibility results via a direct SMS to physician and by placing results on patient’s medical chart. Appropriateness of antimicrobial therapy was determined by two independent infectious disease specialists. Antimicrobial therapy for colonization, too-broad or too-narrow regimen were considered inappropriate.

**Results:**

There were 150 subjects in the pre-implementation group and 150 subjects in the post-implementation group. Approximately half of both groups (pre vs. post; *p-value*) were male (50.7% vs. 48.7%; *p=0.73*) with a comparable mean age (66.14±17.13 vs. 68.57±14.41; *p=0.18*). The three leading causative pathogens were *Escherichia coli* (24.0% vs. 26.7%; *p=0.60*), followed by *Klebsiella pneumoniae* (14.0% vs. 16.7%; *p=0.52*), and *Staphylococcus aureus* (9.3% vs. 10.7%; *p=0.70*). The proportion of appropriate antimicrobial therapy within the first 72 hours was significantly higher in the post-implementation group (22.7% vs. 34.0%; *p=0.03).* Good clinical response was significantly higher in the post-implementation group (74.0% vs. 85.3%; *p=0.02*). Furthermore, the 28-day mortality was comparable between the two groups (24.0% vs. 18.0%; *p=0.20*).

**Conclusion:**

Our study confirmed the positive impact of the Hemo-Alert program on the appropriateness of antimicrobial therapy and treatment response among bacteremic patients.

**Disclosures:**

**All Authors**: No reported disclosures

